# Detecting total immunoglobulins in diverse animal species with a novel split enzymatic assay

**DOI:** 10.1186/s12917-019-2126-z

**Published:** 2019-10-28

**Authors:** Marija Drikic, Steven Olsen, Jeroen De Buck

**Affiliations:** Department of Production Animal Health, Faculty of Veterinary Medicine, University of Calgary, Calgary, Alberta Canada

**Keywords:** Immunoglobulins, Bacterial immunoglobulin binding proteins, Trehalase, STIGA

## Abstract

**Background:**

Total immunolobulin G concentration is a useful, albeit underutilized, diagnostic parameter for health assessments of non-domestic animal species, due to a lack of functional diagnostic tools. Traditional assays, including enzyme-linked immunosorbent assay or radial immunodiffusion, require development of specific reagents (e.g., polyclonal antisera and appropriate protocols) for each animal species, precluding wide and easy adoption in wildlife welfare. As an alternative, bacterial virulence factors able to bind IgGs in antigen-independent manner can be used. To further simplify the diagnostic procedure and increase the number of species recognized by an assay, in this study a recently developed Split Trehalase immunoglobulin assay (STIGA) with bIBPs as a sensing elements was used to detect antibodies in 29 species from 9 orders. Three bacterial immunoglobulin binding proteins (protein G, protein A and protein L) were incorporated into STIGA reagents to increase the number of species recognized.

**Results:**

IgG concentrations were detected through glucose production and produced signals were categorized in 4 categories, from not active to strong signal. Activation was detected in almost all tested animal species, apart from birds. Incorporation of Protein G, Protein A and Protein L allowed detection of IgGs in 62, 15.5 and 6.9% of species with a strong signal, respectively. Assays combining 2 bacterial immunoglobulin binding proteins as sensing element generally gave poorer performance than assays with the same bacterial immunoglobulin binding proteins fused to both trehalase fragments.

**Conclusions:**

STIGA assays have potential to be further developed into an easily adoptable diagnostic test for total amount of IgGs in almost any serum sample, independent of species.

## Background

Determining total amount of immunoglobulin G (IgGs) in clinical samples can be valuable in diagnosing diseases like leukemia, establishing the immune status of an animal found dead or killed by hunters or evaluating passive transfer [1–3]. Still, this diagnostic parameter is not frequently used in evaluating animal health, due to limitations of current diagnostic tools. This is particularly true for wildlife species. Traditional diagnostic tests like ELISA or RID, used to estimate total IgG concentrations, rely on availability of polyclonal antisera or monoclonal antibodies specific for IgGs of targeted species. Furthermore, these tests often require customized protocols for each animal species, making them inadequate for wide adoption and easy adaptation to different species.

To overcome this limitation, indirect ELISAs were developed, with species-specific polyclonal antisera (secondary conjugate) replaced by proteins of bacterial origin able to bind IgGs (bacterial immunoglobulin binding proteins, bIBP) [4–6]. These proteins, considered virulence factors in bacteria, bind constant regions of IgGs (regions not involved in antigen recognition) and therefore are suitable for detection of all IgGs present in sample. Furthermore, they recognize IgGs from multiple animal species [7]. Due to this broad specificity, bIBP are used extensively in antibody purification.

The most frequently used bIBP are protein A (pA), protein G (pG) and protein L (pL) [8]. They differ in species specificity and species affinity, in part due to different binding sites [8, 9]. Protein A (pA), derived from *Staphylococcus aureus*, contains 5 Ig-binding domains which interact with an IgG region at the elbow interface between CH2 and CH3 domain [9–11]. Protein A has a strong affinity for all human IgG subclasses apart from IgG3. Also, it does not interact with IgG from many animal species (e.g. horse, cow or chicken) [12]. Protein G, isolated from streptococcal Lancefield groups C and G, binds to the same IgG area as pA, but has only 3 IgG binding domains [13, 14]. Due to this difference in binding sites, when compared to pA, pG interacts with all human IgG subclasses and with higher affinity [15]. Moreover, pG recognizes IgGs from a broader range of animal species [4]. Still, IgGs of several species recognized by pA are not recognized by pG, making it impossible for pG to fully replace pA. Protein L, from *Peptostreptococcus magnus*, binds to the light (L) chain of IgGs with 4 or 5 binding domains, specifically to the kappa chain, in an antigen-nonspecific manner [16, 17]. Thus, its affinity towards IgG will be conditioned by the quantity of kappa chain present in the IgGs. Protein L has 2 independent IgG binding sites; each interacting with a different region on the light chain with a different affinity constant [18].

Different chimeric proteins containing binding sites from 2 bIBPs broaden the number of species recognized. Protein A/G has been used for purification purposes and in indirect ELISA. This chimeric protein is able to bind to IgGs coming from species recognized by pA and/or pG [19, 20]. Similar research has been done with chimeric proteins LG and LA [21–23], although their species specificity has not been fully investigated.

Recently, a novel detection assay (named Split TreA Immunoglobulin G Assay or STIGA) was developed to detect antibodies in bovine colostrum and calf serum [24]. The assay is based on split trehalase technology [25]. Briefly, trehalase (TreA), an *E. coli* enzyme that catalyzes the conversion of trehalose to glucose [26] was split in 2 non-functional fragments, TreA^N^ (N) and TreA^C^ (C). Each fragment was fused to protein G that acts as a sensor for immunoglobulins. The interaction between pG fused to TreA fragments and the constant region of the immunoglobulins induces dimerization of the 2 TreA fragments (TreA^N^ and TreA^C^) and subsequent reconstitution and reactivation of the TreA. The newly activated TreA converts trehalose to produces glucose that is converted into a colorimetric signal by Glucose Oxidase coupled with Horse radish peroxidase. Split trehalase assay requires specific conditions (250 mM of trehalose, pH 6) for optimal performance as well as specific ratio between the split TreA and the analyte under investigation. Specifically, TreA dimerization efficacy decreases with the concentration of the analyte (IgG) [25]. In the previous study [24], split TreA carrying protein G (STIGA) was applied to quantify the amount of bovine IgGs in colostrum and calf serum. There, the optimal concentration of reagents for this type of assay was established and the new test format (92-well plate format) was developed. Furthermore, the performance of STIGA was compared with the gold standard (Radial Immunodifusion) and the correlation higher than 0.9 was observed demonstrating STIGA’s suitability for quantification of IgGs.

The aim of this study was to modify the existing STIGA assay by incorporating different bIBPs (pG, pA and pL) to act as sensing elements and to evaluate its ability to detect antibodies in a broad range of species.

## Results

### Detection of total IgG levels in various animal species

The STIGA version carrying protein G as sensor (GG assay) produced a strong signal with the majority of animal species tested. The GG assay detected IgGs with a strong signal in 62% of species analysed (18/29), but failed to detect IgGs in 20% (6/29) of species analyzed. The AA assay detected IgGs with a strong signal in 15% (5 spp.) of species and medium to strong signal in 12% (3 spp.) of species. The AA assay detected with a weak signal IgGs in 45% (14 spp.) of species. The LL assaydetected IgGs with a strong signal in only 6.9% (2 spp.) of species, whereas it displayed a weak signal for the majority of species (77.6%; 22 spp.). Assays based on a combination of 2 sensor proteins detected fewer animal species with a strong signal compared to assays containing the same sensor protein. The AL assay failed to detect antibodies in 65% (29 spp.) of species and it detected only 6.9% (2 spp.) of species with a strong signal. GA and GL assays yielded a strong signal in only 19% (10 spp.) and 10.3% (3 spp.) of species, respectively, whereas these 2 assays failed to detect IgGs in 57.9% (11 spp.) and 50% (14 spp.) of species, respectively (Table 1).

**Table 1:**
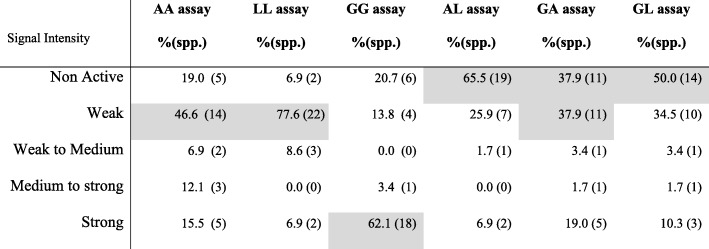
Overall performance of 6 different STIGA assays in 29 different animal species. Percentage and number of species of which the IgG was detected by six different versions of STIGA assays at different intensities

In grey, the predominant performance of a specific STIGA version

### Activation patterns in different animal orders and species

Various versions of STIGA had distinct activation patterns for various animal orders. Aves (Birds) IgGs were only weakly detected with LL assay. Other assays failed to detect antibodies for species in this order. Diprodontia (Marsupials) IgGs were detected weakly by AA, LL and AL assays. Artidactyla (Even-toed ungulates) and Perossidactyla (Odd-toed ungulates) IgGs were strongly detected by a GG assay, but weakly by AA or LL assays. Combination assays also performed weakly in these orders. Proboscidea (Proboscideans) IgGs were mainly detected with medium to strong signals with a GG assay. Carnivora (Carnivores) antibodies were mainly detected with an AA assay, whereas GG and LL assays underperformed in detecting these antibodies. Lagomorpha (Lagomorphs) IgGs were detected by AA, GG and GA assays, whereas assays containing L protein as a sensor failed to detect antibodies in this order. Rodentia (Rodents) IgGs were detected with weak to medium signals by GG, GA and LG assays. Lastly, primate IgGs were efficiently detected with all 6 combinations of split TreA detection assay (Tables 2 and 3). Activation patterns specific to each species used in this study are shown in Table 3.

**Table 2:**
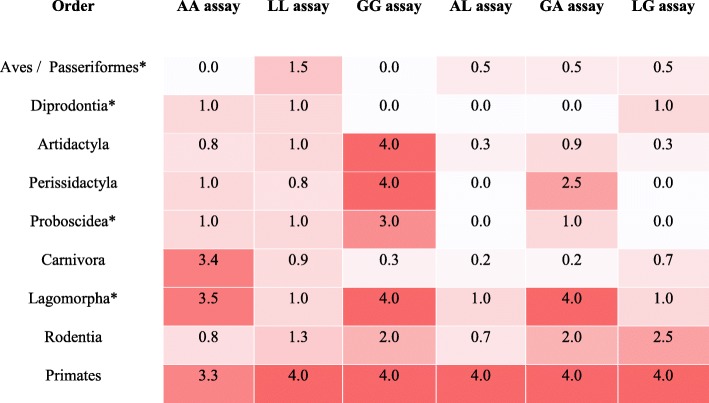
Activation pattern of different versions of STIGA assay in 9 different animal orders

(Red = 4.0 - strong signal; White = 0.0 - Non Active)

*only one animal species tested per order

**Table 3:**
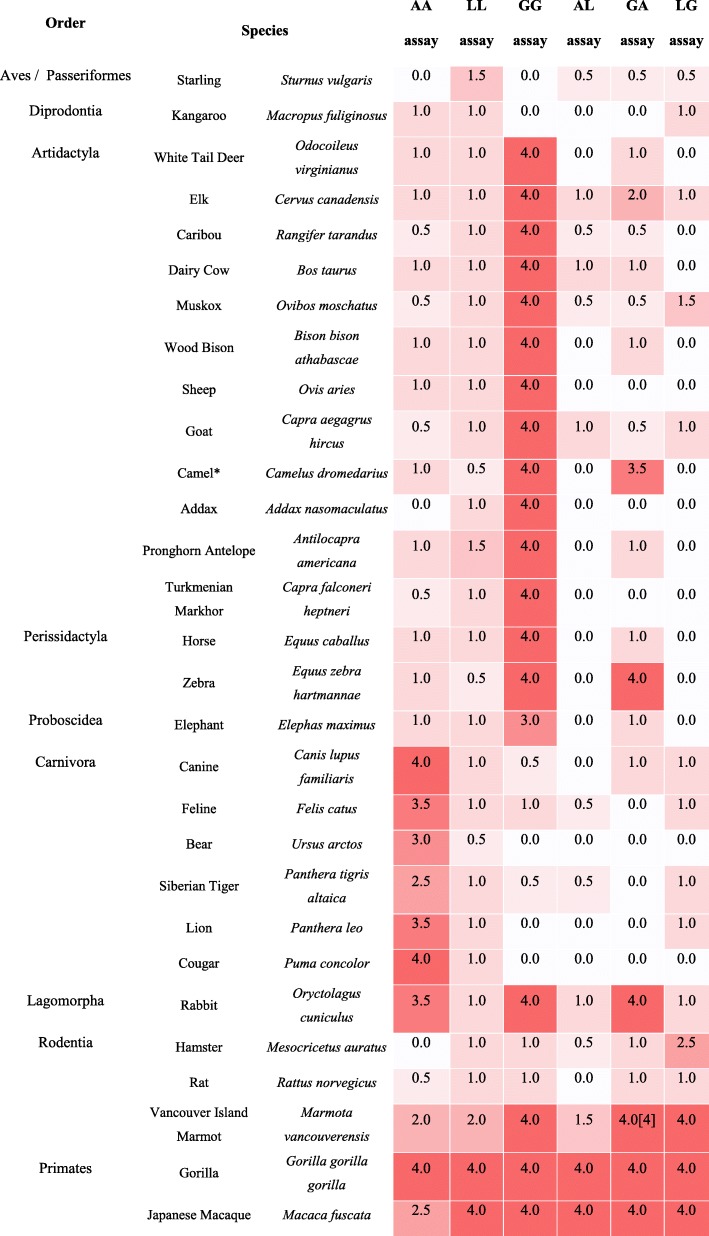
Activation pattern of different versions of STIGA assay in animal species from 9 different animal orders

(Red = 4.0 - strong signal; White = 0.0 - Non Active)

## Discussion

The ability of bIBP to bind to IgGs in a species-independent manner has been used extensively for antibody purification and wildlife antibody ELISA [5, 6, 27]. In this study, bIBP were combined with a novel split TreA detection assay named STIGA to create a total IgG detection assay easily applicable to samples from various animal species. A simple 1-step protocol was applied, without species specific optimization, to efficiently detect IgGs in 29 animal species. Six versions of the modified STIGA assay detected IgGs from almost all animal species examined, except birds and marsupials. Bird antibodies were only detected with an LL assay in a weak manner. This finding confirmed previous reports that bird IgGs (IgY) are not recognized by any bIBP due to their unique structure [12, 15]. Conversely, an inability to detect marsupial IgGs was unexpected, since their IgGs were bound at least by pA [5].

Various STIGA versions had varying detection efficiencies among animal orders, although their detection efficiency stayed constant within an order. Perhaps bIBP have evolved towards binding IgGs from different hosts, although our study design and number of species did not facilitate detailed analyses.

The ability of protein A and protein G to bind antibodies from various animal species was thoroughly investigated in previous studies [3–5]. In a study from 2002, pA and pG were used in a modified ELISA to detect antibodies from 160 zoo animal species representing 7 orders [5]. In general, our STIGA detection efficiency aligned with these findings. In the present study, Carnivore IgGs were detected mainly with an AA assay, whereas Even and Odd-toed ungulate IgGs were mainly detected with a GG assay. Primate IgGs were detected by both bIBP with equal efficiency. Exceptions were marsupial IgGs, which were weakly detected or not detected at all and proboscidean IgGs which were detected with a GG assay but not by an AA assay, contrary to previous findings [5]. Less is known about the ability of protein L to bind IgGs from various wildlife animal species. Regardless, at least 1 species per order was analyzed [17] which could be considered representative for the order, based on low variation within other orders. STIGA detection results confirmed previous studies regarding the ability of pL to bind IgGs. However, the LL assay was less efficient for detection of total IgGs, even though the pL affinity constant for IgGs is high. In that regard, pL is only able to detect the fraction of IgGs with a kappa light chain. Whereas in humans the kappa to lambda light chain ratio is 2:1, in cattle, for example, this ratio is 1:20. Consequently, the LL assay detected primate IgGs with a strong signal, whereas even-toed ungulate IgGs were detected weakly.

STIGA assays containing 2 distinct bIBP had surprisingly low efficiency in detecting IgG, with failure rates ~ 50%. Some exceptions were zebras and camels, where a GA assay had a strong signal, wherea the AA assay did not. Also, in VI marmot, whereas the AA and LL assays had weak signals, the AL assay had a strong signal. In previous studies, chimeric proteins carrying binding sites from 2 bIBP increased the number of species recognized [20], this was not the case when 2 distinct bIBP were used as sensing elements in STIGA. This was most likely failure of our STIGA assay to dimerize. It is probable that binding of 2 STIGA components to different sites on IgGs inhibited dimerization of the reporter components (TreA). This could be due to the improper length of the linkers or to steric hindrance.

Compared to techniques most frequently used for IgG detection, ELISA and RID, the method used in this study is faster, less laborious and does not require additional optimization. Furthermore, this method does not rely on antibody-specific polyclonal antisera essential for ELISA and RID. Reagents for this assay are produced in an inexpensive bacterial recombinant system, eliminating the need for animals in reagent production and reducing costs.

Some of the limitations of this study arose from opportunistic samples used. Details on immune status and age of animals were unknown. Both of these conditions have significant influences on overall quantity of IgGs in the sample and therefore on the outcome of STIGA detection. Too high concentration of IgGs impairs STIGA detection because then the two biosensor fragments are increasingly binding to different IgG molecules and unable to complement. This can be seen in Additional file 1: Figure S1. Too low concentrations result in trehalase activity below the threshold of detection. Furthermore, data on quality of sera was not available. Serum samples tested could have contained compounds (residues of previous treatment) with negative effects on the performance of split TreA assay.

## Conclusions

In conclusion, STIGA assays has been successfully modified by incorporating three different bIBPs and used for detection of IgGs coming from numerous animal species. These results indicate that modified STIGA has the potential to be the start point for development of an easily applicable and inexpensive diagnostic test for total amount of IgGs in serum samples. With further development, appropriate optimization and design and validation of novel sensing elements able to bind IgGs from species not recognized by available IBP, STIGA assays could become applicable wide number of serum samples, coming from various species.

## Methods

### Serum samples

Serum samples were collected from 29 animal species (2 animals/species). Sera were acquired opportunistically from existing frozen collections in research labs at the University of Calgary and at the Calgary Zoo, under veterinary sciences animal care committee (VSACC) protocol AC16–0246 and Calgary Zoo Welfare, Ethics and Research Committee protocol 1,039,994, respectively.

### Strains, plasmids, and other materials

Plasmids encoding for the biosensor used in this study were constructed according to the strategy used previously [25]. In short, 2 TreA fragment sequences, *TreA*^*N*^ (198 bp long) and *TreA*^*C*^ (1368 bp long) were amplified by PCR from the coding sequence of the enzyme TreA. Each gene fragment was then fused at the C-terminal with the coding sequence of 1 of 3 immunoglobulin binding proteins: Protein G (pG) (PBD: 2J52_A), protein A (pA) (PDB: 1HZ5_A) or protein L (pL) (PDB: 1BDC_A) (IDT, Kanata, Canada). All recombinant genes were cloned in pETDuet expression vector (Novagen, Canada) using NcoI and AvrII restriction sites. Purified human IgG (10 mg/ml) used for standard curves was purchased from Sigma-Aldrich (Oakville, Canada).

### Protein purification and lyophilisation

All recombinant proteins were expressed in *E. coli* expression strain BL-21 ΔTreA and purified on Ni-NTA resin (Thermo Fisher Scientific, Ottawa, Canada), as described [25]. In short, the protein expression was induced with 0.5 mM of IPTG (isopropyl-β-D-1-thiogalactopyranoside) (UBP Bio, Aurora, CO) for 3 h at 37 °C. Bacterial pellets expressing recombinant proteins were re-suspended in 6 M guanidinium buffer, lysed by sonication and loaded on equilibrated Ni-NTA resin. Proteins were refolded on resin during washing steps containing gradually decreasing guanidinium-HCl (Sigma-Aldrich, Oakville, Canada) concentrations and eluted in an elution buffer containing 500 mM of imidazole (Sigma-Aldrich, Oakville, Canada). Finally, samples were dialyzed against 1 L of sodium maleate buffer (Sigma-Aldrich, Oakville, Canada) (50 mM, pH 6) with Snakeskin (Fisher Thermo Fisher Scientific, Ottawa, Canada) for 24 h at 25 °C and protein concentration determined by Qubit assay (Thermo Fisher Scientific, Ottawa, Canada).

### Split TreA detection assay for IgGs – modified STIGA assay

**Fig. 1:**
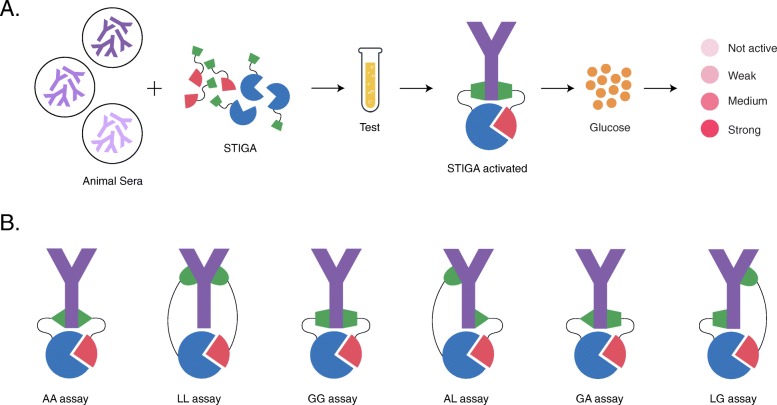
Schematic representation of STIGA assays. **a** Mechanism of activation and signal detection of STIGA assay; **b** Six different versions of STIGA assays. Figure created by MD

Sera from every animal species used in the study was examined with 6 versions of this detection assay (TreA^C^ (C) and TreA^N^ (N) fragments carrying different sensors): 1) N-pA + C-pA (AA assay); 2) N-pL + C-pL (LL assay); 3) N-pG + C-pG (GG assay); 4) N-pA + C-pL (AL assay); 5) N-pG + C-pA (GA assay); 6) N-pL + C-pG (LG assay) (Fig. 1).

Modified STIGA assay was performed with 10 μg of C fragments and 2.85 μg of N fragments (1 to 1 M ratio) in sodium maleate buffer with 250 mM of trehalose (Sigma-Aldrich, Oakville, Canada). Sera were diluted 1 in 100 in sodium maleate buffer (50 mM, pH 6). The dilution factor (DF) was established experimentally (Additional file 1: Figure S1). The glucose concentration was measured with a colorimetric enzymatic assay based on glucose oxidase (2.6 U/mL; Sigma-Aldrich, Oakville, Canada), horseradish peroxidase (0.2 U/mL; Sigma), and O-dianisidine (0.5 mM; Sigma-Aldrich, Oakville, Canada) in sodium maleate buffer (50 mM, pH 6). The final volume of each reaction was 150 μl. All the assays were performed in 96-well plate. Split TreA detection assay reagents were added to the plate; next sera samples were diluted into the reaction. Glucose detecting reagents were added at the end. The reaction was incubated for 90 min at 25 °C. Absorbance (OD) was measured after 90 min in EnSpire multimode plate reader at 450 nm (EnSpire, PerkinElmer, Waltham, MA) [24, 25].

### Data analyses

Background signals caused by glucose in the sera were measured for each serum sample and subtracted from the signal (OD value). All signals (OD values) were converted into a percentage and compared to the signal given by the purified human IgGs used, which was set as maximum (100%) according to the following formula **OD**_**F**_ **= ((OD**_**S**_**-OD**_**B**_**)/OD**_**C**_)*100 (**OD**_**S**_: Optical density of the animal serum with the biosensor (Signal); **OD**_**B**_: Optical density of the animal serum only (Background); **OD**_**C**_: Optical density of purified human antibodies with the biosensor (Positive control)). Signals were split in 4 different categories according to their percentages: Non Active (0), Weak (1), Weak to Medium (2), Medium to Strong (3), Strong (4) (Table 4). Signals coming from the samples belonging to the same species were averaged and presented as a single datum point.

**Table 4:** Signal categories based on the intensity of different split TreA detection assay versions. Four different categories of signal were based on percentages of the signal obtained with human IgG

Signal	Percentage^a^
Non Active (0.0)	< 10%
Weak (1.0)	10–30%
Weak to Medium (2.0)	30–50%
Medium to Strong (3.0)	50–70%
Strong (4.0)	> 70%

*OD*_*S*_ Optical density of the animal serum with the biosensor (Signal), *OD*_*B*_ Optical density of the animal serum only (Background), *OD*_*C*_ Optical density of purified human antibodies with the biosensor (Positive control)

^a^Formula used for the signal intensity calculation: OD_F_ = ((OD_S_-OD_B_)/OD_C_)*100

Animals were grouped according to the order they belong to (Class Aves - Order: Passiformes and Order: Passeriformes (Birds); Class Mammalia: and Order: Diprotodontia (Marsupials), Artiodactyla (Even-toed ungulates), Proboscidea (Proboscideans), Carnivora (Carnivores), Perissodactyla (Odd-toed ungulates), Lagomorpha (Lagomorphs), Rodentia (Rodents) and Primates (Primates)) and signals intensities were examined for each order and each species separately.

## Supplementary information

**Additional file 1: Figure S1.** Effect of human IgG concentration (A; *n* = 3) and serum dilution factor (DF) (B) (DF of 25; *n* = 3) C (DF of 100; *n* = 3); D) (DF of 500; *n* = 3) on STIGA performance in sera samples of three animal species. Trehalase activity and the resulting glucose concentration were measured by GOx-HRP assay after 90 min of incubation at 25 °C.

## Data Availability

The datasets analysed during the current study are available from the corresponding author on reasonable request.

## Abbreviations

bIBP: Bacterial immunoglobulin binding proteins
ELISA: Enzyme-linked immunosorbent assay
IgG: Immunoglobulins G
OD: Optical density
pA: Protein A
pG: Protein G
pL: Protein L
RID: Radial immunodiffusion
STIGA: Split Trehalase IgG quantification assay
