# Supplementary material for: Detecting total immunoglobulins in diverse animal species with a novel split enzymatic assay
Source: BMC Vet Res. 2019 Oct 28;15:374. doi: 10.1186/s12917-019-2126-z (PMC6816151; doi:10.1186/s12917-019-2126-z)
Supplement: Supplementary file 1 — Additional file 1: Figure S1. Effect of human IgG concentration (A; n = 3) and serum dilution factor (DF) (B) (DF of 25; n = 3) C (DF of 100; n = 3); D) (DF of 500; n = 3) on STIGA performance in sera samples of three animal species. Trehalase activity and the resulting glucose concentration were measured by GOx-HRP assay after 90 min of incubation at 25 °C. [file 12917_2019_2126_MOESM1_ESM.docx]

Figure S1.

A)

B)

C)

D)
